# Modulation of D_3_R Splicing, Signaling, and Expression by D_1_R through PKA→PTB Phosphorylation

**DOI:** 10.3390/biomedicines12010206

**Published:** 2024-01-17

**Authors:** Orlando Casados-Delgado, José Arturo Avalos-Fuentes, Manuel Lara-Lozano, Gisela Tovar-Medina, Carla Daniela Florán-Hernández, Karla Gisela Martínez-Nolasco, Hernán Cortes, Ricardo Felix, José Segovia, Benjamín Florán

**Affiliations:** 1 Departamento de Fisiología, Biofísica y Neurociencias, Centro de Investigación y de Estudios Avanzados del Instituto Politécnico Nacional, Mexico City 07360, Mexico; orlando.casados@cinvestav.mx (O.C.-D.); javalos@cinvestav.mx (J.A.A.-F.); manuel.lara@cinvestav.mx (M.L.-L.); gisela.tovar@cinvestav.mx (G.T.-M.); daniela.floran@cinvestav.mx (C.D.F.-H.); karlag.nolasco@cinvestav.mx (K.G.M.-N.); jsegovia@cinvestav.mx (J.S.); 2 Laboratorio de Medicina Genómica, Departamento de Genómica, Instituto Nacional de Rehabilitación Luis Guillermo Ibarra Ibarra, Mexico City 14389, Mexico; hcortes_c@hotmail.com; 3 Departamento de Biología Celular, Centro de Investigación y de Estudios Avanzados del Instituto Politécnico Nacional, Mexico City 07360, Mexico; rfelix@cinvestav.mx

**Keywords:** D1 receptor, D3 receptor splicing, PTB, PKA

## Abstract

The D_1_R and D_3_R receptors functionally and synergistically interact in striatonigral neurons. Dopaminergic denervation turns this interaction antagonistic, which is correlated with a decrement in D_3_nf isoform and an increment in D_3_R membranal expression. The mechanisms of such changes in D_3_R are attributed to the dysregulation of the expression of their isoforms. The cause and mechanism of this phenomenon remain unknown. Dopaminergic denervation produces a decrement in D_1_R and PKA activity; we propose that the lack of phosphorylation of PTB (regulator of alternative splicing) by PKA produces the dysregulation of D_3_R splicing and changes D_3_R functionality. By using in silico analysis, we found that D_3_R mRNA has motifs for PTB binding and, by RIP, co-precipitates with PTB. Moreover, D_1_R activation via PKA promotes PTB phosphorylation. Acute and 5-day D_1_R blockade decreases the expression of D_3_nf mRNA. The 5-day treatment reduces D_3_R, D_3_nf, and PTB protein in the cytoplasm and increases D_3_R in the membrane and PTB in the nucleus. Finally, the blockade of D_1_R mimics the effect of dopaminergic denervation in D_1_R and D_3_R signaling. Thus, our data indicate that through PKA→PTB, D_1_R modulates D_3_R splicing, expression, and signaling, which are altered during D_1_R blockade or the lack of stimulation in dopaminergic denervation.

## 1. Introduction

The co-activation of D3 receptor (D_3_R) and dopamine D1 receptor (D_1_R) results in two possible signaling pathways. In typical signaling, the D_3_R G_i_-mediated effects oppose D_1_R G_s/olf_ effects [1,2,3,4]; in atypical signaling, D_3_R synergizes and potentiates D_1_R effects via a dimeric interaction mediated by G_s/olf_ ([1,5,6,7,8,9]. In the nigral projections from the dorsal striatum, dopaminergic denervation promotes the apparition of D_3_R typical signaling that coexists with the normal atypical version [10]. The D_3_R typical signal (in which D_3_R opposes the D_1_R stimulation of cAMP accumulation and GABA release) masks the normal atypical signaling, with D_3_R potentiating both parameters. This phenomenon was related to an increase in the expression of D_3_R in the membrane [10]. The dopamine D_3_R isoform D_3_nf regulates the canonical D_3_R expression in the membrane [11,12,13,14]. During dopaminergic denervation, decreased D_3_nf isoform expression and protein–protein interaction with D_3_R allows D_3_R to be inserted into the membrane and signal [10,15]. These findings indicate the altered regulation of D_3_R membranal location and its mRNA alternative splicing during dopaminergic denervation, which can explain the appearance of functional D_3_R that opposes D_1_R. However, to date, the mechanism that generates these changes remains unknown. 

Polypyrimidine tract-binding protein, PTB, is a splicing regulator [16]. PTB regulates dopamine D2 receptors (D_2_R) for alternative splicing in heterologous expression systems, decreasing D_2_R_short_ isoform expression [17]. On the other hand, the nuclear location and function of PTB in splicing are regulated by phosphorylation from PKA [18]. When PKA phosphorylates PTB, it is exported from the nucleus, decreasing its function in splicing [18]. With these antecedents in the case of D_1_R and D_3_R interaction in striatonigral neurons, it can be proposed that the lack of D_1_R activation (activator of PKA) during denervation decreased the phosphorylation of PTB that remains in the nucleus, repressing splicing. This may produce a reduced expression of the D_3_nf isoform that lets D_3_R canonical move to the membrane and signal, producing the typical response. Our proposal assumes that PTB also regulates D_3_R splicing. Here, we study this proposal. 

By using in silico analysis, we found that D_3_R mRNA has motifs for PTB binding and, by RIP, co-precipitates with PTB. Moreover, D_1_R activation via PKA promotes PTB phosphorylation. Acute and 5-day blockades of D_1_R decrease the expression of D_3_nf mRNA, which correlates with a decrease in D_3_R, D_3_nf, and PTB protein in the cytoplasm and an increment in D_3_R in the membrane and PTB in the nucleus. Finally, the blockade of D_1_R mimics the effect of dopaminergic denervation, producing the apparition of the D_3_R-mediated typical response. Thus, our data indicate that D_1_R modulates through PKA→PTB, D_3_R splicing, expression, and signaling, which is altered during D_1_R blockade or the lack of stimulation through dopaminergic denervation. 

## 2. Materials and Methods

### 2.1. In Silico Analysis

We evaluated the presence of splicing regulatory motifs for the deleted sequence of 98 nucleotides that were previously reported for D_3_nf production in D_3_R mRNA using in silico analysis [11,19]. We used the Human Splicing Finder program, version 3.1. We searched for exon and intron regions, GC motifs for 5’ and GA for 3’ splicing sites, branch points (CURAY sequence), and ISS and ESS motifs on polypyrimidine tracts (TY). Delimited sequences were the sixth exon and the two flanking introns with 300 up and down nucleotides that were analyzed. We compared the literature reports for PTB motifs in different splicing sites with respect to the obtained results from our analysis to test whether PTB is a potential regulator of D_3_R splicing at this exon.

### 2.2. Animals

Male Wistar rats (200–250 g) housed together (five per cage) with food and water ad libitum and kept under a natural light cycle were used throughout. All procedures followed the National Institutes of Health Guide for Care and Use of Laboratory Animals. They were approved by the Institutional Animal Care Committee of the CINVESTAV, making all efforts to minimize animal suffering.

### 2.3. SCH23390 Treatment

Rats were injected in two ways; according to the experiment, some rats received a single dose of 0.5 mg/kg i.p. of D_1_R antagonist SCH 23390 and were sacrificed at 6 or 12 h after injection. Others received 0.5 mg/kg of SCH23390 or saline twice daily (every 12 h) for 5 days and, on the 6th day, were sacrificed for experiments. Rats for mRNA immunoprecipitation and PTB phosphorylation assay did not receive SCH 23390 treatment. 

### 2.4. Whole Striatum Homogenates and Brain Slice Preparation

After rapid decapitation, the brains were removed and immersed in ice-cold artificial cerebrospinal fluid (aCSF) using the following composition (mM): 118.25 NaCl, 1.75 KCl, 1 MgSO_4_, 1.25 KH_2_PO_4_, 25 NaHCO_3_, 2 CaCl_2_, and 10 D-glucose. A rapid dissection of the whole striatum was performed, then the tissue was immersed in aCSF and processed for WB experiments. The cAMP accumulation and PTB phosphorylation experiments were performed in brain slices. The brain was glued to a metal cube mounted on a Petri dish filled with ice-cold aCSF, and brain slices (300 µm thick) containing the striatum were obtained with a vibroslicer (Campden Inc., Cambridge, UK). The slices were transferred to cold slides, and under a stereoscopic microscope, the striatum was microdissected and used in different experiments. The atlas of Paxinos and Watson (2006) [20] was utilized to identify individual nuclei. The slices or whole striatum tissue obtained from the two striata of each rat were pooled and used separately in each experiment so that *n* = 1 represents the data obtained from the two striatum of one rat. 

### 2.5. Cellular Fractionation Procedures

For the WBs of D_3_R, D_3_nf, and PTB in subcellular fractions enriched with the nucleus, cytoplasm, and cell membranes, we used the method modified from Heydorn [21,22]. The whole striatal nuclei of three to five male Wistar rats (8 to 12 weeks old, weighing approximately 220 g) were used. Once dissected, the tissue was weighed and homogenized in seven volumes (*w*/*v*) of 0.32 M sucrose/5 mM HEPES pH 7.4 with protease inhibitors. The homogenate was centrifuged at 800× *g* for 10 min at 4 °C. The resulting precipitate (P1) is the crude nuclear fraction, resuspended in 0.32 M sucrose/5 mM HEPES pH 7.4 with protease inhibitors added; this was stored at −20/−70 °C until its use. The supernatant (S1) was centrifuged at 9200× *g* for 20 min at 4 °C. The precipitate 2 (P2) and supernatant 2 (S2) were obtained. S2 was centrifuged at 105,000*× g* for 90 min at 4 °C, and the supernatant 3 (S3) corresponding to the cytoplasmic fraction was obtained and stored. The precipitate corresponding to the microsomes was discarded. P2 was resuspended for lysis in I0 volumes of 5 mM HEPES pH 7.4 on ice for 30 min. Then, it was centrifuged at 25,000× *g* for 20 min at 4 °C, producing precipitate 3 (P3), and the supernatant containing the post-synaptic vesicles was discarded. P3 was resuspended in a small volume of 0.32 M sucrose/5 mM HEPES pH 7.4 and carefully placed on a stepwise gradient of 0.32, 0.9, and 1.2 M sucrose, respectively, which was centrifuged at 83,000*× g* for 120 min at 4 °C. Two bands were obtained from the previous step, and the interface between the 0.32 and 0.9 sucrose gradient, corresponding to myelin, was discarded. The second interface, between the 0.9 and 1.2 M sucrose gradient, corresponds to the membrane fraction, which was carefully extracted, added with protease inhibitors, and stored for later use.

Cellular fractionation was carried out according to [23,24] for RIP procedures to obtain the enriched nuclear fraction. In brief, striatal tissue was homogenized with 8 mL of cold lysis buffer (HEPES 20 mM, KCl 125 mM, MgCl2 4 mM, NP40 0.05%, one tablet of cOMPLETE (Cat. # 11873580001, Roche, Basel, Switzerland, Sigma-Aldrich, St. Louis, MO, USA) previously solved in 50 mL buffer, 40 μL of RNaseOUT (Cat # 10777-019 Invitrogene. Thermofisher Scientific, Waltham, MA, USA) and 80 μL of Ditiotreitol (DTT), pH 8). The homogenate was stored for 24 h at −80 °C to promote cell lysis. Then, it was centrifuged at 100*× g* for 5 min at 4 °C to remove cell debris. Then, another centrifugation was performed at 900*× g* for 10 min at 4 °C to separate the cytoplasmic and nuclear fractions. The pellet corresponding to the nuclear fraction was washed with 1 mL of sucrose buffer (Tris-HCl 20 mM, NaCl 60 mM, KCl 15 mM, Sucrose 0.34 M, two tablets of Complete previously solved in 20 mL buffer, 5 μL of RNaseOUT, 10 μL of DTT, pH 7.65). The pellet was resuspended with 2 mL of sucrose buffer with 10 μL of RNaseOUT, 20 μL of DTT, and 800 μL of high-salt buffer (Tris-HCl 20 mM, EDTA 0.2mM, Glycerol 25%, NaCl 900 mM, MgCl2 1.5 mM, half of a tablet of cOMPLETE previously solved in 5 mL buffer, 4 μL RNaseOUT and 8 μL DTT, pH 7.65) to promote nuclear membrane rupture; this was incubated in ice for 30 min, reversing every 5 min. For DNA digestion, 4.6 mL of sucrose buffer was added with 25 μL of RNaseOUT and 46 μL of DTT, supplemented with 7.5 μL of CaCl2 1M. Next, 75 μL of DNAsase (Cat # 18068015 DNAsase I, Invitrogen. Thermofisher Scientific) was added and incubated for 15 min at 37 °C, inverting the sample every 5 min. After this time, 60 μL of 0.5M EDTA was added. For soluble nuclear fraction collection, the sample was centrifuged at 16,000*× g* for 20 min at 4 °C, and the supernatant was transferred to a new cold tube.

### 2.6. PTB Phosphorylation Assay

In vitro phosphorylation assay was performed as described by Loya López (2020). Striatum (300 μM thick slices) were incubated for 30 min with the D_1_R agonist SKF 38393 (1 μM), the D1R antagonist SCH 23390 (100 nM), or PKA blocker H89 (10 μM), or a combination as indicated. After incubation, the slices were homogenized and centrifugated to obtain nuclei and cytoplasm, purified according to [25], and then separated by electrophoresis in 10% acrylamide gels. Gels were fixed in 50% methanol and 10% acetic acid overnight. Then, they were washed with ultrapure water three times and stained with Pro-Q Diamond phosphoprotein gel staining (Invitrogen) for 90 min. Gels were de-stained with a solution containing 20% acetonitrile, 50 mM sodium acetate, pH 4.0, 30 min, three times, and, finally, the phosphoprotein bands were detected using a GelDoc EZ from BioRad. The resulting bands were analyzed by using densitometry using ImageJ software, Version 1.46 (NIH, Bethesda, MD, USA).

### 2.7. mRNA Immunoprecipitation (RIP)

First, we prepared magnetic beads with PTB antibodies. A total of 100 μL of lysis buffer with 130 μL of magnetic beads and 5 μg of antibody against PTB were mixed. Then, they were incubated for 1 h at 4 °C to promote binding and were centrifuged at 5000*× g* for 15 s. Six washes were performed with 1 mL of lysis buffer to resuspend the beads in 900 μL of cold lysis buffer with 4.5 μL RNaseOUT and 9 μL DTT. 

The supernatant of the nuclear fraction was transferred to the magnetic beads already attached to the antibody and was left to incubate in rotation at 4 °C overnight. The next day, four washes were performed with 500 μL cold lysis buffer, 2.5 μL RNaseOUT, and 5 μL DTT. The beads were resuspended with 100 μL lysis buffer, 0.5 μL of RNaseOUT, and 1 μL DTT. In order to elute the RNA bound to the beads, the protein was digested by adding 1.5 μL of Proteinase K solution (Cat 4333793 Invitrogene Thermofisher Scientific) 20 mg/mL) and incubated for 30 min at 55 °C in constant agitation. Finally, the supernatant was collected.

Total RNA was extracted by the TRIzol method. A total of 1 mL of TRIzol (Invitrogen, 15596026) was added to the supernatant collected from RNA immunoprecipitation. The sample was incubated for 5 min at room temperature, and then 200 μL of chloroform was added. The sample was shaken by inversion, incubated for 3 min at room temperature, and centrifuged for 15 min at 12,000 rpm at 4 °C. The aqueous phase containing the RNA was transferred to a new tube. Next, 500 μL of 100% isopropanol was added and incubated for 24 h at −20 °C. The next day, the sample was centrifuged for 10 min at 12,000 rpm at 4 °C, and the supernatant was removed. Subsequently, the RNA precipitate was washed two times with 1 mL of 75% ethanol. The supernatant was discarded, and the RNA pellet was dried for 10 min. The pellet was resuspended in 20 μL of RNase-free water (Gibco, Waltham, MA, USA) and incubated in a bath at 60 °C for 10 min.

Finally, cDNA was synthesized. The reaction was prepared with 2 μg of RNA, 1 μL of Random Primers (Random Primers Cat. 48190011 Invitrogene. Thermofisher Scientific), and 2 μL of 10 mM dNTPs. The reaction was incubated at 65 °C for 5 min and then placed on ice. Subsequently, 4 μL of 5X FS buffer, 2 μL of DTT 100 mM, and 1 μL of RNaseOUT were added to leave the sample incubating for 2 min at 37 °C. Then, 1 μL of the enzyme M-MLV RT (Thermofisher, 28025013) was added and incubated for 10 min at 25 °C. It was then incubated at 37 °C for 50 min. Finally, the sample was incubated at 70 °C for 15 min to inactivate the enzyme and was stored on ice.

### 2.8. Western Blot 

For the Western Blot (WB) assay, 50 µg of protein (to detect PTB, D_3_nf, and D_3_R from coprecipitate samples) from subcellular fractionation were resolved by SDS-PAGE, transferred onto nitrocellulose membranes, and blotted for 2 h at room temperature in Tris-buffered saline containing 0.1% Tween 20 and 10% nonfat powdered milk. Membranes were incubated overnight at 4 °C with either antibody against PTB (1:1000), D_3_R (1:1000), or D_3_nf (1:1000). Polyclonal antibodies for D_3_R and D_3_nf were obtained from Santa Cruz Biotechnology Inc. numbers: D3DR (H-50): sc-9114, and D_3_nf (C-16): sc-9184. PTB antibody was purchased from Invitrogen, Thermo Fisher Scientific Inc., PTBP1 monoclonal antibody 32-4800. Antibodies were detected by chemiluminescence (ECL Plus Amersham) with HRP-conjugated secondary antibodies (1:20,000 dilution for antibodies against primary antibodies.

### 2.9. qRT-PCR Determinations

Following the treatment periods, the animals were euthanized, and both striatal regions were carefully dissected from brain slices, as described in Section 2.4. The dissected striatal tissues were collected in tubes containing 200 µL of TRIzol on ice. Subsequently, the tissues were macerated to achieve homogenization, and TRIzol was added until a final volume of 1 mL was reached. Total RNA extraction was then performed, as previously mentioned in Section 2.7. After total RNA purification, the sample’s purity and total RNA integrity were assessed before cDNA synthesis.

For tissue cDNA synthesis, 5 µg of total RNA was used in a final volume of 8 µL. This RNA was treated with 1 µL of DNase I and 1 µL of DNase I Buffer (10X). The reaction was incubated for 15 min at room temperature, and it was stopped by a heat shock and the addition of 1 µL of EDTA. In order to the previous reaction, 1 µL of Oligo dT (500 µg/µL) was added, followed by incubation at 65 °C for 5 min and 1 min on ice. The RT protocol was carried out using the parameters reported in Section 2.7.

For the qRT-PCR reactions from RIP cDNA and tissue cDNA, pre-designed probes from Thermofisher were used to evaluate the expression of the D_3_R gene (Rn00567568_m1) and D_3_nf gene (AIT96RG), and for the tissue samples, the Hprt1 gene (Rn01527840_m) was employed as an endogenous control [10]. The reaction mixtures contained 5 µL of 2x Master Mix (Applied Biosystems, Waltham, MA, USA), 2 µL of the sample, 0.5 µL of the respective probe, and 2.5 µL of water. The thermocycling conditions were as follows: an initial denaturation cycle at 94 °C for 5 min, followed by 55 cycles with two steps, 94 °C for 15 s, and 60 °C for 60 s. These qPCR reactions were carried out using the Bio-Rad CFX96 real-time equipment. Data were analyzed by using 2^−ΔCt^ and 2^−ΔΔCt^ calculations [26]. The fold change was obtained by comparing the SCH 23390-treated rats vs. the saline group and the ratio D_3_nf/D_3_R + D_3_nf to evaluate the relative expression of the isoforms in the saline vs. SCH 23390-treated condition.

### 2.10. cAMP Accumulation 

We carried out [^3^H]cAMP accumulation assays according to the method described by Rangel-Barajas et al. [27]. Striatal slices obtained from one rat were pooled and incubated with [^3^H]-Adenine (130 nM) for 1 h at 37 °C. After this, the radioactive samples was washed thrice and then suspended in aSCF with 3-isobutyl-1-methylxanthine (IBMX) 1 mM. Incubation with dopamine receptor drugs was continued for 15 min and stopped by adding 100 μL of ice-cold trichloroacetic acid (15%) containing non-labeled ATP (2.5 mM) and cAMP (4.5 mM). N-ethylene maleimide (NEM) [10] was incubated with dopaminergic drugs to block Gi protein signaling. After 20 min on ice, the supernatant was loaded onto Dowex 50W-X4 (300 μL per column). A fraction containing [^3^H]-ATP was eluted with 3 mL of distilled water. A second eluent obtained with 5 mL of distilled water was directly loaded onto neutral alumina columns. Alumina columns were finally eluted with 4 mL of 50 mM Tris–HCl buffer (pH 7.4) to obtain [^3^H]-cAMP. ATP and cAMP eluents were transferred to vials, and radioactivity was determined by scintillation counting. The results were expressed as the ratio of [^3^H]- cAMP × 100/[^3^H]-cAMP + [^3^H]-ATP and then as a percent of the control condition.

### 2.11. Drugs

3-isobutyl-1-methylxanthine, IBMX; R(+)-7-Chloro-8-hydroxy-3-methyl-1-phenyl-2,3,4,5-tetrahydro-1H-3-benzazepine hydrochloride, SCH 23390; (+)-(4aR,10bR)-3,4,4a,10b-Tetrahydro-4-propyl-2H,5H-[1]benzopyrano [4,3-b]-1,4-oxazin-9-ol hydrochloride, PD 128,907; (±)-1-Phenyl-2,3,4,5-tetrahydro-(1H)-3-benzazepine-7,8-diol hydrochloride, SKF38393; were purchase from Merk KGaA, Mexico. 4′-Acetyl-N-[4-[4-(2-methoxyphenyl)-1-piperazinyl] butyl]-[1,1′-biphenyl]-4-carboxamide, GR103691 was obtained from Tocris Bioscience, CTR Scientific, Mexico.

Radiochemical: Adenine, [2,8-3H]^−^, >97%, 1 mCi (37MBq), [^3^H] Adenine; Aminobutyric Acid (GABA) γ-[2,3-3H(N)]^−^, Specific Activity: 25–40 Ci (925 GBq-1.48 TBq)/mmoL, 1 mCi (37 MBq) was purchased from Perkin Elmer (Waltham, MA, USA).

### 2.12. Data Analysis

Data are expressed as mean ± SEM. All data were analyzed using Graph Pad Software (San Diego, CA, USA), version 9.4.1. For the differences in three or more experimental conditions running in parallel, a one-way ANOVA combined with Tukey’s test was used to compare the differences in the same experiment. An unpaired *t*-test was employed to compare the two experimental conditions from different experiments or only two experimental conditions from the same experiment.

## 3. Results

### 3.1. In Silico Motifs for Splicing in the mRNA of D_3_R and Co-Immunoprecipitation with PTB

Silico analysis of the sixth exon of D_3_R mRNA showed that the 98 deleted nucleotides in D_3_nf are characterized as an intronic region. The GC motif 5’ position with a 78.48 score and GA motif 3’ position with a 73.04 score in the 98-nucleotide region are donor and acceptor regions; moreover, we found two branch points scores of 68.88 and 86.16, and finally, a critical ISS in the 26 position on a TY tract that has a score of 80.18. Furthermore, multiple motifs with high silencer activity were found (see Supplementary Material).

**Figure 1:**
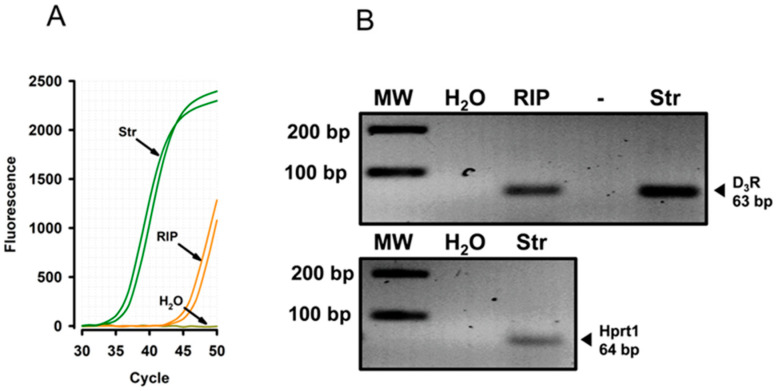
D_3_R mRNA co-precipitates with PTB. (**A**) A qRT-PCR amplification curve for the D_3_R gene from the extract of PTB immunoprecipitated (RIP) and the cDNA from striatal tissue (Str). (**B**) Agarose gel electrophoresis from the amplified cDNA recovered from the qRT-PCR.

In order to assess whether the PTB protein binds to D_3_R mRNA, we conducted an RIP (RNA immunoprecipitation) procedure. After the RIP procedure, the presence of the D_3_R transcript was evaluated through qRT-PCR (see Section 2). The amplification of the D_3_R transcript indicates that D_3_R mRNA co-precipitates with PTB (Figure 1A). In order to confirm that the results of the qRT-PCR were not a nonspecific product, we evaluated the molecular weight of qRT-PCR products by agarose gel electrophoresis. We used cDNA from striatum tissue as a control. As observed, the molecular size of the amplicons from the RIP or striatum tissue presents the same molecular weight, corroborating the findings (Figure 1B).

### 3.2. D_1_R Activation Phosphorylates PTB

**Figure 2:**
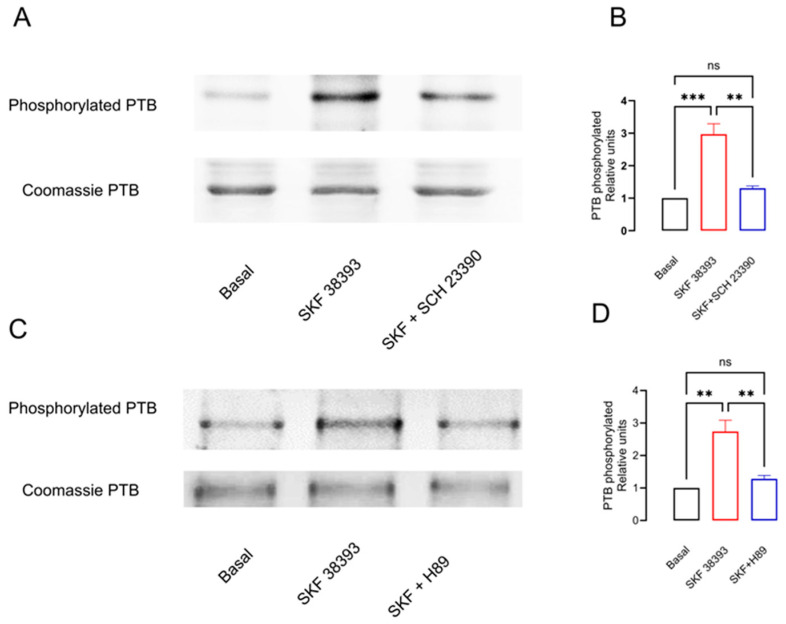
Activation of D_1_R by SKF 38393 phosphorylates PTB by PKA. (**A**,**C**) Representative blots of PTB phosphorylation in homogenates from striatal slices treated with the D_1_R agonist SKF 38393 and the D_1_R antagonist SCH 23390 or the PKA blocker H89. (**B**,**D**) The densitometry analysis from three blots. ** *p* < 0.01; *** *p* < 0.001; ns: not significant differences. (**B**) F = 30.96, *p* = 0.0007; (**D**) F = 19.86, *p* = 0.0023, One-way ANOVA, followed by Tukey’s test, *n* = 3.

In order to test the phosphorylation of PTB promoted by D_1_R activation via PKA, we incubated striatal slices for 15 min with the D_1_R agonist SKF 38393 (1μM) [28] or SKF 38393 in the presence of the D_1_R selective receptor antagonist SCH 23390 (100 nM) [29] or the PKA blocker H89 (10μM) [30], and we determined the PTB phosphorylation level. Figure 2B,D show that D_1_R stimulation increased the basal phosphorylation levels of PTB by almost two times when compared to the basal state (Basal 1 vs. SKF38393 2.98 ± 0.32, F = 1.74 (2,6), *p* < 0.001, *n* = 3 determinations, ANOVA, followed by Tukey’s test). The addition of the antagonist SCH 23390 prevents this effect (Basal 1 vs. SKF38393+ SCH23390 1.30 ± 0.07, F = 1.74 (2,6), *p* = 0.52 ns, *n* = 3 determinations, ANOVA, followed by Tukey’s test). Similarly, the blockade of PKA with H89 prevents the SKF 38393 phosphorylation of PTB (Basal 1 vs. SKF38393+ H89 1.28 ± 0.10, F = 1.01 (2,6), *p* = 0.63 ns, *n* = 3 determinations, ANOVA, followed by Tukey’s test). Figure 2A,C show the representative blots from the three experiments.

### 3.3. D_1_R Blockade Decreases D_3_nf mRNA, Protein Expression, and Cytoplasmic Location

**Figure 3:**
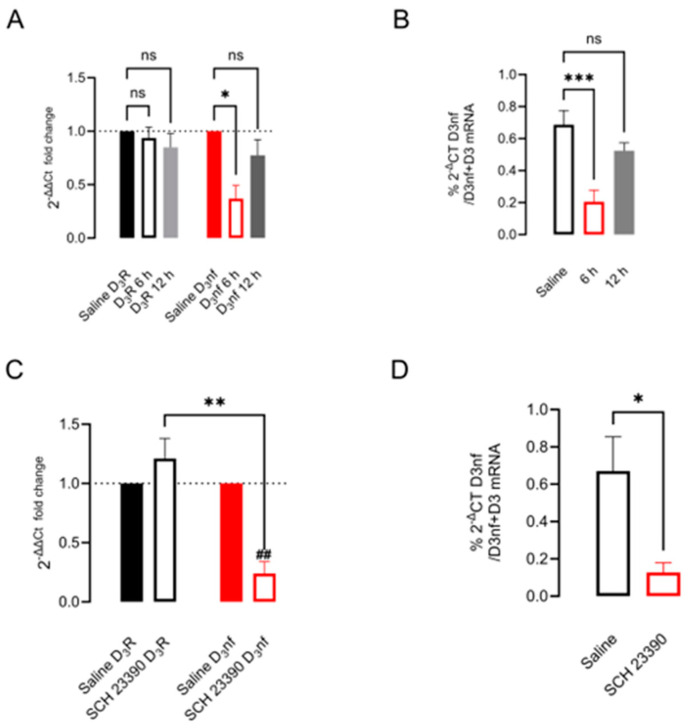
D_1_R receptor blockade with SCH 23390 decreases D_3_nf mRNA expression and relative expression concerning D_3_R mRNA. (**A**) The effect of a single dose of the D_1_R antagonist SCH 23390 with respect to saline in striatal D_3_nf and D_3_R mRNAs at 6 and 12 h after injection. (**B**) The D_3_nf/D_3_R+D_3_nf ratio of the relative expression of the isoforms. (**C**,**D**) The same determinations after a 5-day two-dose antagonist treatment. * *p* < 0.05; ** *p* < 0.01; *** *p* < 0.001; ns: not significant; ^##^ *p* < 0.01 with respect to the indicated group. (**A**) F = 4.29, *p* = 0.0056; (**B**) F = 10.25, *p* = 0.0021; (**C**) F = 18.72, *p* < 0.0001. One-way ANOVA, followed by Tukey’s test, *n* = 3–6 determinations, (**D**) *t* = 4.91, df = 6, unpaired *t*-test *n* = 4.

We tested if the lack of stimulation of D_1_R changes D_3_nf expression in a similar way to what is observed in dopaminergic denervation. First, in normal rats, we blockaded D_1_R via a single injection of SCH 23390 0.5 mgr./kg i.p.; after 6 and 12 h later, we determined the striatal expression of D_3_R and D_3_nf mRNA via qRT-PCR. Figure 3A shows a decrement of nearly 65% in the 2^-ΔΔCT^-fold change in D_3_nf mRNA 6 h post-injection, with a tendency to recover to saline control values 12 h post-injection (*p* = 0.014 at 6 h; *p* = 0.785 at 12 h; *n* = 6, ANOVA, followed by Tukey’s test); no significant changes in 2^-ΔΔCT^-fold change for D_3_R mRNA were found (Figure 3A). When evaluating the relative expression of D_3_nf mRNA compared to D_3_+D_3_nf in treated and saline control samples, a significant decrement was observed at 6 h. However, the mRNA levels tended to recover 12 h post-treatment (Figure 3B: ratio control 0.68 ± 0.08 vs. SCH 23390-treated, 6 h, 0.20 ± 0.07, *p* = 0.0007 vs. 12 h, 0.49 ± 0.06, ns, *n* = 6; ANOVA, followed by Tukey’s test).

**Figure 4:**
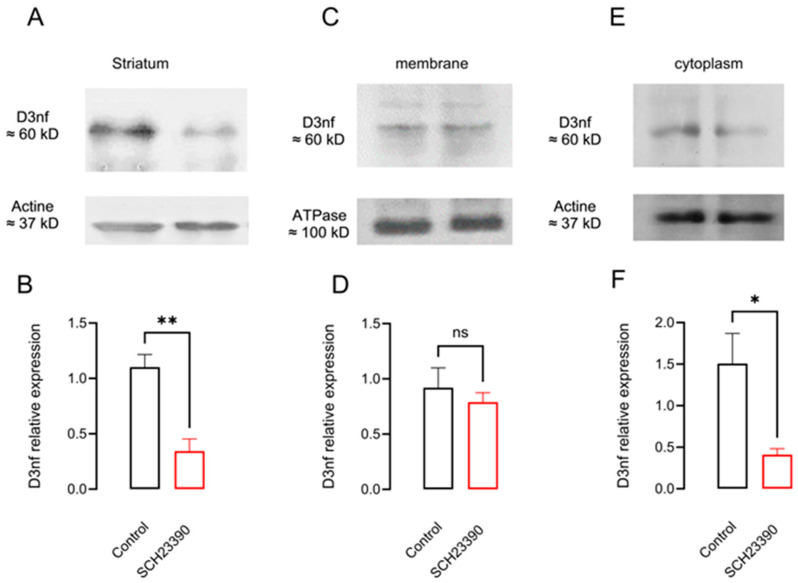
D_1_R blockade with SCH 23390 decreases striatal D_3_nf protein expression in the cytoplasm. (**A**,**C**,**E**) WB from D_3_nf protein from total striatal homogenates and protein fraction from striatal membranes or cytoplasm, respectively. (**B**,**D**,**F**) The densitometry analysis. * *p* < 0.05; ** *p* < 0.01; ns: not significant: (**B**), *t* = 4.81, df = 6, *n* = 4; (**D**), *t* = 0.65, df = 4, *n* = 3; (**F**), *t* = 2.96, df = 4, *n* = 3. Unpaired *t*-test.

The effect of 5 days of D_1_R blockade from a twice-a-day injection of D_1_R selective antagonist SCH 23390 on the mRNA and protein expression of D_3_nf in the whole striatum and the membranal and cytoplasmic subcellular fractions is shown in Figure 3C,D and Figure 4. In Figure 3C, it can be observed that D_1_R blockade decreases the expression of the mRNA of D_3_nf by nearly 75% with respect to the saline condition since the 2^−ΔΔCT^-fold change decreases to 0.24 ± 0.10 (*p* = 0.005, *n* = 4, *t*-test). In Figure 3D, it can be observed that the percent ratio of D_3_nf/D_3_nf+D_3_R also decreases significantly in the treated group (ratio control 0.67 ± 0.18 vs. SCH 23390-treated 0.12 ± 0.05, *p* = 0.021, *t* = 3.3, *n* = 4, and unpaired *t*-test). As expected from the mRNA decrement observed, an important decrement in D_3_nf protein expression was observed in the whole striatal tissue (Figure 4A,B) (D_3_nf relative expression with respect to actine: control 1.10 ± 0.11 vs. SCH23390-treated 0.34 ± 0.10, *p* = 0.003, *t* = 4.81, *n* = 4, and unpaired *t*-test). The decrement in protein expression occurs mainly in the cytoplasm, as can be observed in Figure 4E,F (D_3_nf relative expression with respect to the actine control 1.57 ± 0.36 vs. SCH23390-treated 0.41 ± 0.07, *p* = 0.041, *t* = 2.95, *n* = 3, and unpaired *t*-test); but this is not the case in the protein located in the cell membrane (Figure 4E,F; D_3_nf relative expression respect ATPase control 0.912 ± 0.18 vs. SCH23390-treated 0.79 ± 0.08, *p* = 0.92, *t* = 0.54, *n* = 3. Unpaired *t*-test). Figure 4A,C,E represent typical Western blots.

### 3.4. D_1_R Blockade Did Not Modify D_3_R mRNA but Increased Its Membrane Location

**Figure 5:**
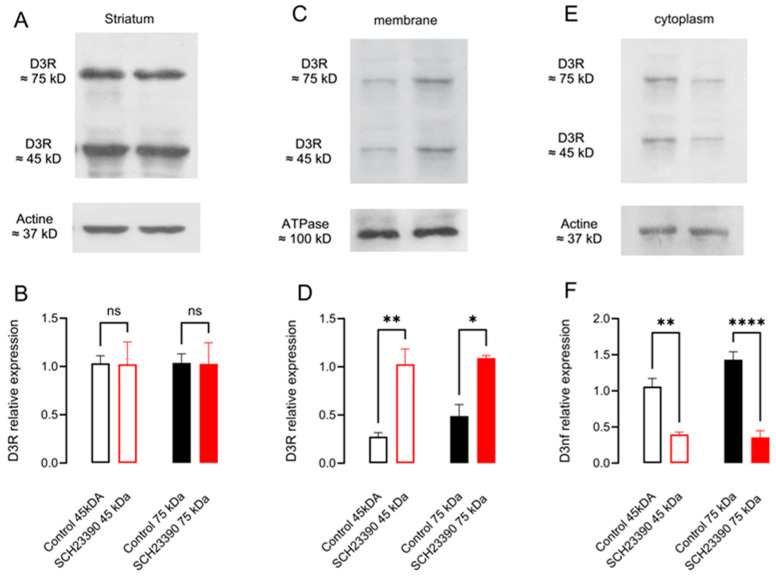
D_1_R blockade with SCH 23390 did not change striatal D_3_R protein expression but increased it in the membrane. (**A**,**C**,**E**) WB from D_3_R protein from total striatal homogenates and protein fraction from striatal membranes or cytoplasm, respectively; the 45 kD bands represent the D_3_R monomeric forms and the 75 kD bands represent the dimeric forms. (**B**,**D**,**F**) The densitometry analysis. * *p* < 0.05; ** *p* < 0.01; **** *p* < 0.0001; ns: not significant. (**B**), F = 0.001, *p* > 0.999; (**D**), F = 15.22, *p* = 0.0011; (**C**), F = 31.73, *p* < 0.0001. One-way ANOVA, followed by Tukey *n* = 3.

We also studied if D_1_R blockade affects the expression of D_3_R canonical isoform. In Figure 3A, it can be observed that no statistically significant change in D_3_R mRNA expression occurs in the striatum of the SCH 23390-treated rats since a 2^-ΔΔCT^-fold change concerning the saline condition did not differ from the expected value of 1 (1.31 ± 0.37 vs. 1, *p* = 0.46, *n* = 4; one sample *t*-test). Additionally, SCH 23390 did not affect D_3_R protein levels when evaluating the whole striatal protein content for both the monomers (45 kDa) and dimers (75 kDa) of the receptor [10,31,32] (Figure 5B: D_3_R 45 kDa relative expression with respect to actine control 1.03 ± 0.08 vs. SCH23390-treated 1.023 ± 0.23, *p* = 0.97, *t* = 0.04 and D_3_R 75 kDa relative expression control 1.036 ± 0.09 vs. SCH 23390-treated 1.027 ± 0.22, *p* = 0.97, ns, *n* = 3. Unpaired *t*-test). Interestingly, D_3_R protein expression increases in the membrane and decreases in the cytoplasm (Figure 5D: D_3_R 45 kDa relative expression with respect to ATPase, control 0.27 ± 0.04 vs. SCH23390-treated 1.027 ± 0.16, *p* = 0.01, *t* = 4.55 and D_3_R 75 kDa relative expression control 0.49 ± 0.12 vs. SCH 23390-treated 1.09 ± 0.02, *p* = 0.008; Figure 5F: D_3_R 45 kDa relative expression respect actine control 1.06 ± 0.11 vs. SCH23390-treated 0.396 ± 0.033, *p* = 0.005, *t* = 5.59 and D_3_R 75 kDa relative expression control 1.43 ± 0.11 vs. SCH 23390-treated 0.357 ± 0.09, *p* = 0.001 *n* = 3. Unpaired *t*-test). Figure 5A,C,E represent typical Western blots.

### 3.5. D_1_R Blockade Increased PTB Expression in the Nucleus

**Figure 6:**
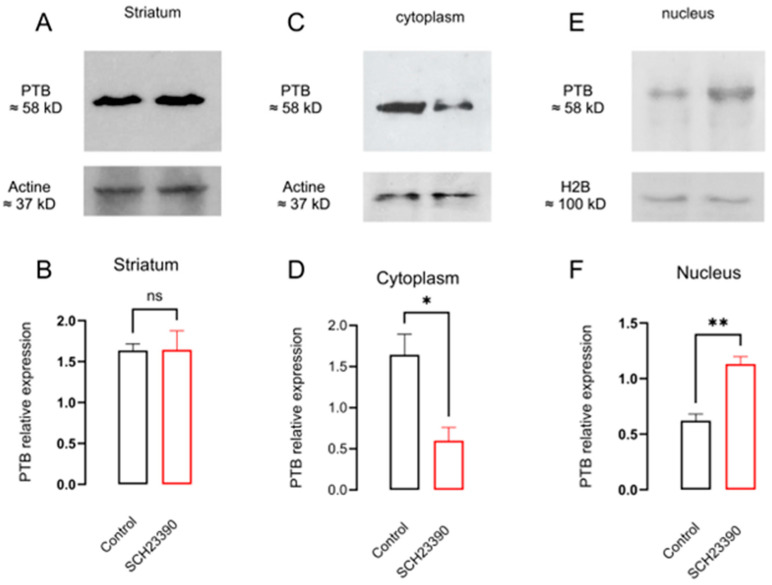
D_1_R blockade with SCH 23390 did not change striatal PTB protein expression but decreased it in the cytoplasm and increased it in the nucleus. (**A**,**C**,**E**) WB for D_3_R protein from total striatal homogenates and protein fraction from striatal cytoplasm or nucleus, respectively. (**B**,**D**,**F**) The densitometry analysis. * *p* < 0.05; ** *p* < 0.01; ns: not significant differences, (**B**), *t* = 0.03, df = 4, *n* = 3; (**D**), *t* = 3.5, df = 4, *n* = 3; (**F**), *t* = 5.7, df = 4, *n* = 3. Unpaired *t*-test.

The effects on PTB protein expression via D_1_R blockade are shown in Figure 6. First, SCH 23390 treatment did not modify the protein expression in whole striatum tissue (Figure 6B: PTB relative expression with respect to actine control 1.64 ± 0.08 vs. SCH 23390-treated 1.64 ± 0.23, *p* = 0.98, *t* = 0.03, *n* = 3. Unpaired *t*-test). Nevertheless, it decreases protein in the cytoplasm and increases it in the nuclear fraction (Figure 6D: PTB relative expression with respect to actine control in the cytoplasm 1.64 ± 0.25 vs. SCH 23390-treated 0.59 ± 0.16, *p* = 0.025, *t* = 3.50, *n* = 3; Figure 6F: PTB relative expression with respect to actine control in nucleus 0.62 ± 0.06 vs. SCH 23390-treated 1.13 ± 0.06, *p* = 0.004, *t* = 5.70, *n* = 3. Unpaired *t*-test). Figure 6A,C,E represent representative Western blots.

### 3.6. Blockade of D_1_R Produces D_3_R Typical Signaling and Masks the Atypical One

**Figure 7:**
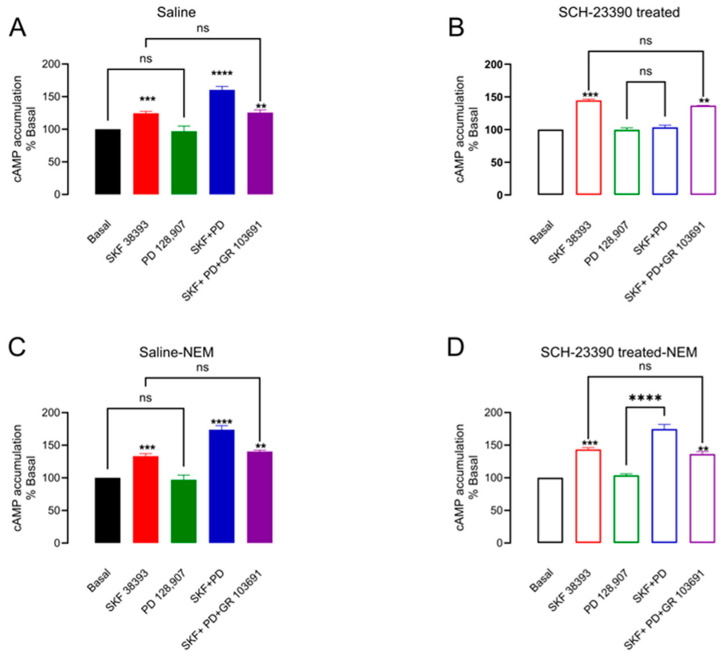
D_1_R blockade with SCH 23390 produces the typical D_3_R response that masks the atypical response. (**A**,**B**) cAMP accumulation experiments in control striatal slices and 5-day SCH23390 treatment. (**A**) D_1_R activation by SKF38393 increases cAMP, and the D_3_R activation by PD 128,907 potentiates it; (**B**) The response of D_1_R activation is prevented by D_3_R activation. (**C**,**D**) are shown in the same experiments but in the presence of Gi protein coupling blocked by NEM, which restores the D_3_R atypical response in (**D**). ** *p* < 0.01, *** *p* < 0.001*;* ns: not significant. (**A**) F = 37.79, **** *p* < 0.0001; (**B**) F = 116.7, *p* < 0.0001; (**C**) F = 53.55, *p* < 0.0001; (**D**) F = 76.88, *p* < 0.0001. One-way ANOVA, followed by Tukey *n* = 3.

Finally, we studied if D_1_R blockade produces the same changes in D_3_R signaling as dopaminergic denervation [8,10]. We studied the stimulation of cAMP accumulation via D_1_R and D_1_R+D_3_R co-activation in the striatal slices of the saline and SCH 23390-treated rats. D_1_R was stimulated with SKF38393 (1 μM) and D_3_R, with a PD of 128,907 (100 nM) [33]. In Figure 7A, the atypical response of D_3_R activation on the D_1_R stimulation of cAMP accumulation can be observed (potentiation), whereas the D_3_R agonist itself did not have an effect (Figure 7A: cAMP accumulation SKF38393 124 ± 3% vs. SKF38393+PD128,907 160 ± 5%, *p* = 0.0014, *n* = 6). In the SCH 23390-treated group, D_3_R activation antagonized the D_1_R stimulation of cAMP accumulation, which signifies the atypical response (Figure 7B: cAMP accumulation SKF38393 144 ± 1.85% vs. SKF38393+PD128,907 103 ± 3.83%, *p* < 0.0001, *n* = 3).

In order to test if the D_3_R atypical response masks the typical one, we blockaded Gi protein signaling by performing experiments in the presence of NEM (100 μM) [34]. In Figure 7C, it can be observed that in the saline-treated rats, the atypical response is preserved, even in the presence of NEM. On the contrary, in the SCH 23390-treated rats, the same atypical response as in the saline condition is observed (see Figure 7D).

## 4. Discussion

Several patho-physiological processes are related to changes in the splicing of D_3_R, which modify its signal and location [35]. Here, for the first time, we found evidence of the role of D_1_R, PKA, and PTB in regulating D_3_R splicing in striatal neurons. Our data indicate that D_1_R regulates D_3_R alternative splicing through PTB phosphorylation via PKA. They also suggest that PTB is a splicing regulator of D_3_R mRNA that promotes the expression of the D_3_nf isoform, which, as was previously suggested, regulates the membranal expression of the canonical receptor [14]. Finally, this mechanism impacts the functional effect of D_1_R and D_3_R interaction in striatal neurons.

### 4.1. PTB Modulates D_3_R mRNA Alternative Splicing and Produces D_3_nf Expression

Initially, the mRNA 98-nucleotide sequence deleted in D_3_nf mRNA was characterized in the human gene [11,36,37], but some differences in the mRNA of *Rattus Norvegicus* can be observed in the intron, based on the ensemble genome browser (see supplementary data), which indicates only an 87% analogy between both. In order to test the presence of PTB motifs in the rat sequence, we performed an in silico analysis of the reported rat sequence using Human Splicing Finder 3.1 software [11] (see Supplementary Material). In our analysis and agreement with the Schmauss group [11,36,37], the GC and GA sequences found are potential 3’ and 5’ splicing sites; GC is the more frequently atypic donor motif, and the GA site is considered a non-canonic acceptor site with a frequency of 1:19 [38]. In addition, our analysis shows that the deleted sequence has two potential ramification points with a high score. Thus, all in silico analysis data support the idea that the sequence is an atypical intronic region in the mRNA of the rat, as was reported for humans. Sironi’s motifs analysis of human deleted mRNA sequence indicates 15 silencer sequences with scores of more than 60, from which nine matched the rat mRNA [39]. The literature-reported union motifs for PTB: CTC [16], TCC [40], CCT [16], CTTA [41], and CCCT [16] are present in the deleted sequence as well as the CCTGC sequence of the polypyrimidine tract [42,43]. The presence of the 3’ splicing site near the PT tract, the presence of several Sironi’s motifs, the literature-reported repressor motifs for PTB, and our RIP data (Figure 1) strongly suggest that PTB can bind the deleted sequence in the mRNA and inhibit the intronic definition [44]. More research is needed to elucidate the specific sites involved in the intronic definition via PTB.

### 4.2. D_1_R Modulation of PTB Phosphorylation, Function, and Cellular Location

Our data indicate that D_1_R activation by SKF 38393 promotes the robust phosphorylation of PTB (almost 150%, Figure 2), which is D_1_R-specific and PKA-dependent. Knowing that PKA is the main regulator of PTB phosphorylation, and according to its nuclear location [18,45], this result is expected. Moreover, to our knowledge, this is the first report in a naïve system in which D_1_R phosphorylates PTB and modifies its splicing repressor activity during its blockade, as well as changing its cellular location and affecting the mRNA and protein expression of the D_3_nf isoform.

The effects of D_1_R on PTB functionality can be observed better in the long-time receptor blockade with SCH23390. That is because it produces chronic effects in the expression, location, and function of proteins processed by the regulated mRNA splicing, which is the case in dopaminergic denervation [10,15] and the 5-day blockade of D_1_R (this report). In the striatum, D_1_R activates adenylyl cyclase and incrementally effects cAMP production, which activates PKA [46]. When PKA is activated, the catalytic subunit translocates to the nucleus and phosphorylates PTB at Ser 16 [45]; then, PTB relocates from the nucleus to the cytoplasm, decreasing the repression of splicing and increasing the expression of isoforms: [47] in this case, D_3_nf. On the contrary, based on this proposal regarding the regulation of PTB by D_1_R, when D_1_R is chronically blockaded, it maintains decreased PKA activity, whereby no displacement of PTB occurs, remaining in the nucleus (Figure 6), repressing D_3_R splicing, and, as a consequence, decreasing D_3_nf isoform mRNA and protein expression (Figure 3).

Our model of a 5-day D_1_R blockade has two main characteristics that are required for producing the changes observed: D_1_R blockade and decreased PKA activity. It has been shown that injected SCH 23390 (at a dose of 0.1 mg/kg i.p.) has a peak concentration in the striatum for upwards of 3 h, whereas in plasma, it decreases over 1 h. This long-lasting concentration in the brain correlates with a decrement in Vmax for adenylyl cyclase and the locomotor activity (up to 80%) 12 h after injection concerning the control [48]. Additionally, the changes in locomotor activity stimulated by D_1_R in the striatum correlate with changes in PKA activity [49]. These antecedents indicate that in our model, the two doses a day of 0.5 mgr./kg of SCH 23390 for 5 days would produce a persistent blockade of D_1_R activity since this dose of antagonist occupies 95% of receptors [50]. This long-time receptor occupancy decreases cAMP formation via adenylyl cyclase and decreases PKA activity. Although D_1_R blockade could increase D_1_R membranal receptors [51], they remain occupied by SCH 23390. The need for the 5-day treatment is because, for the blockade of D_1_R using a single dose, the decrease in D_3_nf expression at 6 h tends to recover at 12 h, as can be seen in Figure 3A,B; thus, no significant accumulated changes in the expression of proteins was evident. On the other hand, studying the regulation of the splicing of D_3_R mRNA is important because the changes in D_3_R and D_3_nf isoform expression have important repercussions in chronic diseases in which D_1_R is not active or overactivated [10,14,35].

Interestingly, D_1_R blockade did not modify D_3_R mRNA or protein (Figure 3 and Figure 5), except for its cellular location. One can expect that an increment in canonical D_3_R could compensate for a decrease in the expression of D_3_nf, but that is not the case. It should be considered that the relative expression of D_3_nf mRNA concerning D_3_R mRNA in a normal condition is 0.6%, and this decreases up to 0.1% during blockade (Figure 3). The amount of D_3_R mRNA is too high compared to D_3_nf mRNA; thus, no significant change can be observed in our experimental conditions if such compensation occurs.

A fact that has claimed attention is the amount of D_3_nf protein expressed, which is almost comparable to that of D_3_R protein; this is essential since it has a physiological role: regulating D_3_R expression into the membrane [14,35]. How can this relatively low mRNA produce a significant amount of protein? This requires more experimental research; however, PTB has also been noted to be involved in maintaining mature mRNA stability and ribosomal transduction into protein [47,52]. Thus, PTB may be the factor that ensures enough levels of D_3_nf protein. On the other hand, in neurons, PTB is a factor capable of regulating the level of genic expression by inhibiting the exportation of incompletely spliced mRNA from the cytoplasm, a factor that triggers the nuclear degradation of the messenger (in this case, of D_3_R mRNA [53]), thus equalizing the translation into the protein of the D_3_R and D_3_nf isoforms [10,15]. More research is needed to clarify these points.

Finally, the changes in the location of the D_3_R protein are related to the decrement in the D_3_nf isoform since D_3_nf dimerizes the D_3_R protein, retaining it in the cytoplasm ([10,14]); the decrement in the expression of D_3_nf promotes D_3_R membranal location (Figure 5) [10]. In summary, our data indicate that through PTB phosphorylation, D_1_R regulates the expression of mRNA and the protein location of the PTB, D_3_R, and D_3_nf isoforms.

### 4.3. D_1_R Regulation of D_3_R Splicing Modifies Its Function

The central hypothesis for the role of D_3_nf in D_1_R and D_3_R interaction comes from Ritchand’s research, which proposes that the D_3_nf isoform regulates D_3_R membranal expression [14]. In an antagonistic interaction, the D_3_R opposes D_1_R effects, and by sequestering the canonical D_3_R into the cytoplasm, D_3_nf modulates dopamine’s effect on the receptors that favor the action in the D_1_R. According to a condition in which the D_3_nf isoform decreases, more D_3_R moves to the membrane and efficiently antagonizes D_1_R activity. Thus, Ritchand’s proposal explains the increased membrane expression of D_3_R observed in dopaminergic denervation and in the 5-day blockade of D_1_R [10,15,54], except when there is no favor for D_1_R activity because the normal response between D_1_R and D_3_R are synergistic. The most important finding in this work is that D_1_R regulates the expression of D_3_R in the membrane by regulating splicing. On the other hand, in the nucleus accumbens, D_3_R receptor activation also regulates D_3_R and D_3_nf mRNAs expression [55]; thus, in dopaminergic denervation, it is expected that the lack of stimulation of D_3_R will reduce both D_3_R and D_3_nf messengers. However, in our previous report in the denervated striatum, this is not the case; only D_3_nf messenger is decreased, and the fact that D_1_R blockade mimics the effects of denervation supports the notion of the role of D_1_R in regulating the membranal expression of D_3_R and decrements in the D_3_nf isoform [10,54]. It is interesting to note that the function of the nucleus accumbens regarding D_3_R is antagonistic with respect to D_1_R; thus, increments in D_3_R expression will produce less D_1_R activity in terms of competition for the dopamine available [14] in the case of the dorsal striatum when D_3_R is retained in the cytoplasm and favors the activity of the D_1_R-D_3_R dimmers; this regulation marks substantial differences in the function of D_3_R in both regions of the striatum.

**Figure 8:**
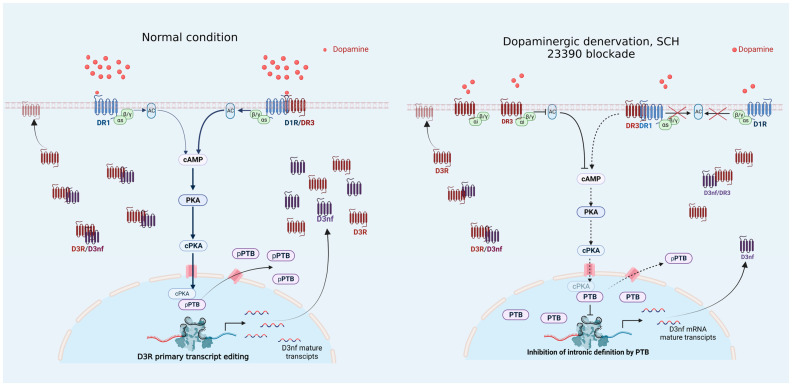
Schematic drawing illustrating the D_1_R or D_1_R/D_3_R signaling to regulate the PTB modulation of the splicing of D_3_R in a normal condition and D_1_R blockade or dopaminergic denervation.

In the dorsal striatum, D_1_R and D_3_R interact in proportion to medium-sized spine striatal neurons and their projections [6,7,8,9]. Therein, D_3_R potentiates D_1_R effects (atypical signaling) in a dimeric interaction; thus, during denervation or 5 days of SCH 23390 treatment, the new membranal functional D_3_R appears, which opposes (typical signaling) D_1_R-D_3_R synergistic effects [10]. This new membrane receptor is functionally coupled to the Gi protein, masking atypical signaling since Gi protein blockade unmasks a synergistic relationship that is not lost (Figure 7). In agreement with this, no significant changes in D_3_R mRNA occur (Figure 2) [10], nor do they happen in the number of D_1_R-D_3_R mRNA-expressing neurons during denervation [56,57]. These observations imply that in a normal condition, not all D_3_R protein is located in the membrane; that is, an amount of protein is retained in the cytoplasm by the D_3_nf isoform and the action regulated by D_1_R (Figure 5). Why does a neuron produce D_3_R protein and keep it in the cytoplasm but not in the membrane? This requires research; however, since D_1_R-D_3_R participates in regulating motor behavior [58], the regulation of membranal D_3_R expression is critical since its activation will antagonize the motor effects of the dimmer [10]. In this sense, it can be proposed that regulating D_3_R splicing is a normal mechanism to control the adequate function of the D_1_R-D_3_R dimmer. In summary, the data support the notion of D_1_R regulating D_3_R splicing and function, which is altered during dopaminergic denervation by a lack of activity of dopamine on D_1_R, leading to a modified functional response (Figure 8).

## 5. Conclusions

Our data indicate that through PKA→PTB, D_1_R modulates D_3_R splicing, expression, and signaling, which is altered during D_1_R blockade or a lack of stimulation, as is the case in dopaminergic denervation. The dysregulation of D_3_R expression implies the co-existence of synergistic and antagonistic effects on D_1_R function, altering the behavioral response to their activation.

Since several patho-physiological processes are related to the changes in the splicing of D_3_R that modify its signal and location [35], knowledge of those factors that determine the regulation of the splicing is essential to understand the changes in the pathology of Parkinson’s disease and to propose of new therapeutical approach.

## Data Availability

Data are available upon request due to privacy/ethical considerations.

## Supplementary Materials

The following supporting information can be downloaded at: https://www.mdpi.com/article/10.3390/biomedicines12010206/s1.
