# Supplementary material for: Modulation of D3R Splicing, Signaling, and Expression by D1R through PKA→PTB Phosphorylation
Source: Biomedicines. 2024 Jan 17;12(1):206. doi: 10.3390/biomedicines12010206 (PMC10813448; doi:10.3390/biomedicines12010206)
Supplement: Supplementary file 1 [file biomedicines-2812667-supplementary.pdf › biomedicines-2812667-supplementary.pdf]

Supplementary material

5´ GUCCCACCAU AGCGCCCAAG CUCAGCUUAG AAGUUCGAAA ACUCAGCAAU GGCAGAUUAU CGACAUCUUU GAAGCUGGGG CCCCUGCAAC CUCGGGGA 3´
5´ GCCCCACCAU GGCACCCAAG CUCAGCUUAG AGGUUCGAAA ACUCAGCAAC GGCAGGUUAU CCACGUCCCU GAGGCUGGGG CCCCUGCAGC CUCGGGGA 3´

87% percent of homology between the *Homo Sapiens* and *Rattus Norvegicus* gene that encodes to D3 receptor in the 98-nucleotides deleted in D_3nf. DRD3 Homo Sapiens gene (DRD3 ENSG00000151577) in black, Drd3 Rattus Norvegicus gene (Drd3 ENSRNOG00000060806) in red. The green boxes show the differences between the nucleotide sequences.

Link in database:

Human
https://www.ensembl.org/Homo_sapiens/Gene/Summary?db=core;g=ENSG00000151577;r=3:114127580-114199407

Rat
http://www.ensembl.org/Rattus_norvegicus/Gene/Summary?g=ENSRNOG00000060806;r=11:56879689-56940596

Sequence Position	cDNA Position	Sironi's motif	Silencer motif	Value 0-100 (variation)
-98	c.861-98	Motif 3 - TCTCCCAA	tcccacca	61.63
-97	c.861-97	Motif 3 - TCTCCCAA	cccaccat	60.04
-93	c.861-93	Motif 1 - CTAGAGGT	ccatagcg	62.38
-91	c.861-91	Motif 1 - CTAGAGGT	atagcgcc	62.12
-88	c.861-88	Motif 3 - TCTCCCAA	gcgcccaa	82.54
-73	c.861-73	Motif 1 - CTAGAGGT	ttagaagt	73.45
-27	c.861-27	Motif 2 - [T/G]G[T/A]GGGG	agctgggg	72.83
-26	c.861-26	Motif 2 - [T/G]G[T/A]GGGG	gctggggc	80.18
-24	c.861-24	Motif 2 - [T/G]G[T/A]GGGG	tggggccc	60.69
-17	c.861-17	Motif 3 - TCTCCCAA	cctgcaac	61.79
-9	c.861-9	Motif 1 - CTAGAGGT	ctcgggga	60.33
-9	c.861-9	Motif 2 - [T/G]G[T/A]GGGG	ctcgggga	60.84
-7	c.861-7	Motif 2 - [T/G]G[T/A]GGGG	cggggagT	60.84
-4	c.861-4	Motif 2 - [T/G]G[T/A]GGGG	ggagTGCC	67.10

Potential splicing silencers Sironi motifs present in the 98-nucleotide sequence deleted in the D_3nf receptor in the Homo Sapiens gene, characterized by Human Splicing Finder 3.1. In yellow, the sequences that coincide with Rattus *norvegicus* are observed.

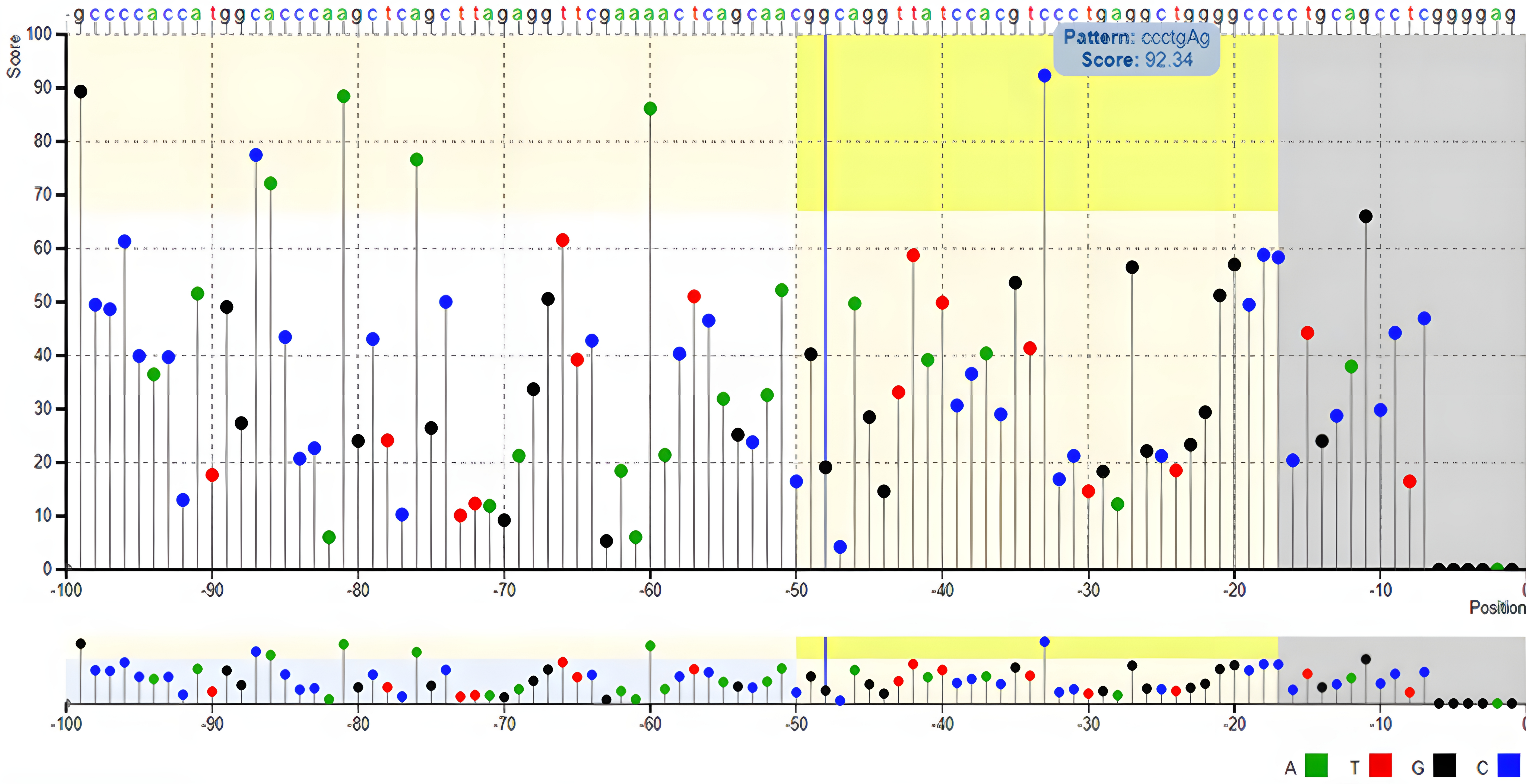

Heptamer position	Branch Point motif	Consensus Value (0-100)
-33	ccctgAg	92.34
-60	aactcAg	86.16

Light green colored boxes indicate motifs with consensus value superior to 67. Dark green colored boxes indicate motifs consensus value superior to 70 and having a good position according to the AG dinucleotide exclusion zone from Gooding et al.

In silico characterization of the potential branch points present in the sequence of the 98 nucleotides deleted in the D3nf receptor of *Rattus Norvegicus* with a high score.

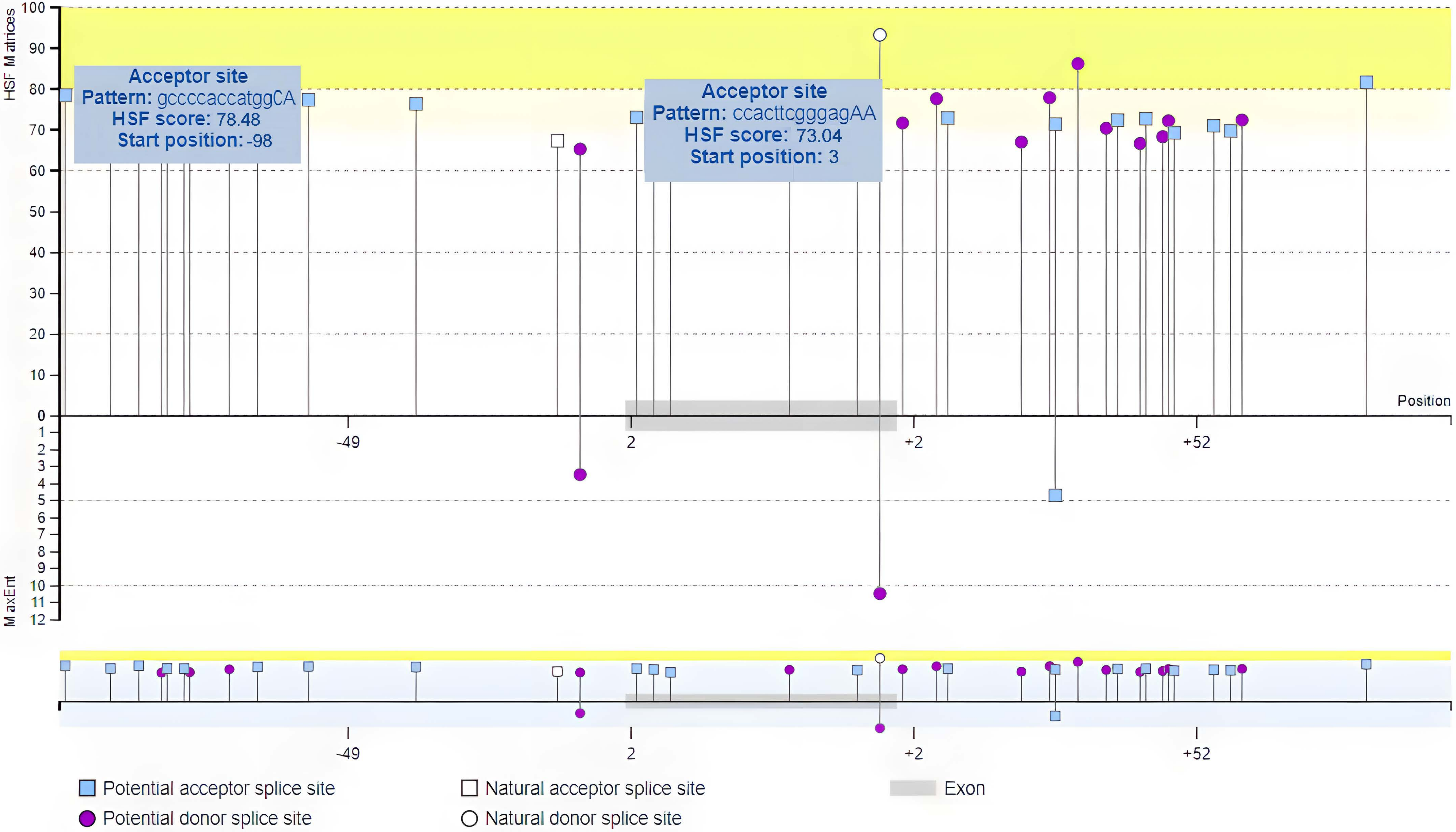

HSF Matrices
MaxEnt
Acceptor site
Pattern: gccccaccatggCA
HSF score: 78.48
Start position: -98
Acceptor site
Pattern: ccacttcgggagAA
HSF score: 73.04
Start position: 3
Position
-49
2
+2
+52
Potential acceptor splice site
Potential donor splice site
Natural acceptor splice site
Natural donor splice site
Exon
